# Revelations from the Nematode *Caenorhabditis elegans* on the Complex Interplay of Metal Toxicological Mechanisms

**DOI:** 10.1155/2011/895236

**Published:** 2011-08-17

**Authors:** Ebany J. Martinez-Finley, Michael Aschner

**Affiliations:** ^1^Division of Pediatric Clinical Pharmacology and Toxicology, Vanderbilt University Medical Center, Nashville, USA; ^2^Center in Molecular Toxicology, Vanderbilt University Medical Center, Nashville, TN, USA; ^3^Center for Molecular Neuroscience, Vanderbilt University Medical Center, Nashville, TN, USA; ^4^The Kennedy Center for Research on Human Development, Vanderbilt University Medical Center, Nashville, TN, USA; ^5^Division of Pediatric Toxicology, Vanderbilt University Medical Center, 11425 MRB IV, 2215-B Garland Ave., Nashville, TN 37232-0414, USA

## Abstract

Metals have been definitively linked to a number of disease states. Due to the widespread existence of metals in our environment from both natural and anthropogenic sources, understanding the mechanisms of their cellular detoxification is of upmost importance. Organisms have evolved cellular detoxification systems including glutathione, metallothioneins, pumps and transporters, and heat shock proteins to regulate intracellular metal levels. The model organism, *Caenorhabditis elegans* (*C. elegans*), contains these systems and provides several advantages for deciphering the mechanisms of metal detoxification. This review provides a brief summary of contemporary literature on the various mechanisms involved in the cellular detoxification of metals, specifically, antimony, arsenic, cadmium, copper, manganese, mercury, and depleted uranium using the *C. elegans* model system for investigation and analysis.

## 1. Introduction

Exposure to metals remains a persistent toxicological concern. The accumulation of metals in the environment, stemming from their origination in the earth's crust, as well as from anthropogenic sources, creates the potential for significant human exposures and subsequent health hazards. Deleterious metal-induced health effects, including carcinogenesis and neurodegeneration, have been reported in all body systems, with exposure stemming from multiple sources, including contact with contaminated food, water, air, or soil. The particular metals considered in this review, antimony, arsenic, cadmium, manganese, mercury, silver, and uranium, are among the classes of essential nutrients, as is the case with copper and manganese, as well as the nonessential, naturally occurring metals, such as arsenic, cadmium, and mercury, all of which can induce toxicity depending on the concentration level and exposure duration. Over time, organisms have developed protective mechanisms to deal with metal exposure, most of which function in one of three ways: (1) decreasing the uptake of the metal, (2) stimulating the expulsion of the metal, or (3) activating the organism's general stress response mechanisms. Metals can disrupt homeostasis by generating oxidative stress, inhibiting enzyme activity, impairing DNA repair, and disrupting protein binding and normal cellular function, including proliferation, cell cycle progression, and apoptosis [1–4].

Elucidating the mechanism(s) of metal detoxification has been difficult due to the complexity of mammalian systems and the reductionist approach inherent to cell culture systems. The model organism, *Caenorhabditis elegans (C. elegans), *offers the advantage of an *in vivo* system that is less complex than the mammalian system while still sharing high homology. *C. elegans* possess ~60%–80% of human genes [5] and contain conserved regulatory proteins [6–8]. 

This soil nematode has been used in a number of toxicity studies due to its well-characterized genetic, physiological, molecular, and developmental stages. Some of the advantages afforded by the *C. elegans* model system are small size (~1.5 mm adult), short lifespan (~3 weeks), and rapid lifecycle (~3 days) [8–10]. At adulthood, a single *C. elegans* hermaphrodite is capable of producing ~300 progeny. *C. elegans* are hermaphrodites, but approximately 1% of *C. elegans* are male, allowing for genetic experimentation [11]. The nematode's small genome and relative anatomical simplicity (less than 1000 cells) contribute to the appeal of this model system for genetic manipulation [11]. In addition, the use of RNA interference (RNAi) and chromosomal deletion in worms has provided valuable information regarding the increased sensitivity of mutant strains to metal toxicity [12–14]. Maintenance of nematode strains is relatively simple; they grow on bacteria-seeded plates and can be maintained at 20°C [15]. Strains can also be frozen indefinitely, easily allowing for the accumulation of large stocks of worms [11]. *C. elegans *provide the researcher with a uniquely powerful model, as the worm's translucent body allows for the *in vivo* visualization of fluorescently labeled individual cells and proteins [16]. Accordingly, the *in vivo C. elegans* model system is especially valuable for the investigation of metal detoxification and is particularly amenable for examining gene-environment interactions, albeit with a few considerations to take into account (Table 1). Several toxicity endpoints are readily detected and well documented in the nematode, including mortality, lifespan, reproduction, and feeding [17–19]. Acute toxicity can also be assessed in the nematode through behavioral endpoints, such as locomotive behavior, head thrashing, body bending, and other basic movements [20–24]. Recently, the role of *C. elegans* as a biomonitor in environmental risk assessment has also been explored [25–28]. 

Several cellular systems such as the glutathione (GSH), metallothioneins (MTs), heat shock proteins (HSPs), as well as various pumps and transporters work in concert to detoxify and excrete metals. It remains to be established whether the knockdown or overload of one detoxifying system upregulates other compensatory mechanisms. In this review, we offer a brief summary of the ways in which the *C. elegans* model has shed novel insights on the various mechanisms of metal detoxification. Metals considered herein are antimony (Sb), arsenic (As), cadmium (Cd), lead (Pb), mercury (Hg), silver (Ag), and uranium (U).

## 2. Glutathione

**Figure 1:**
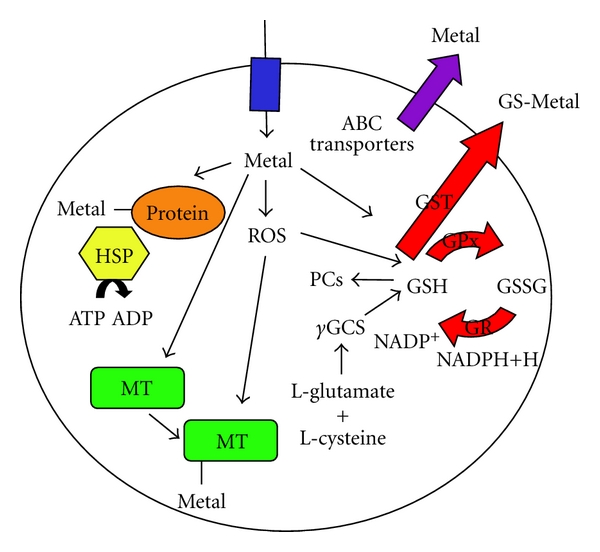
Metal detoxification systems. GSH is converted to GSSG upon exposure to ROS. GR converts GSSG back to GSH while converting NADPH to NADP+. *γ*GCS is the rate-limiting enzyme in GSH synthesis. GSTs assist with the conjugation of GSH to the metal for excretion from the system. Additionally, GSH is known to be protective against metal-generated ROS by binding free radicals. PCs are thiol-rich peptides that can complex with metals and act as chelators. MTs can directly bind and sequester the toxicant and act as antioxidants. ATP-binding cassette (ABC) transporters contribute to heavy metal tolerance by facilitating the excretion of metals, including metals that are conjugated to GS−. HSP70s are ATP-binding proteins that convert ATP to ADP and bind to metals and other proteins, thereby inactivating them and preventing aggregation.

Glutathione (GSH) is a cysteine-containing tripeptide, consisting of glutamic acid, cysteine, and glycine and is found in most life forms. GSH possesses antioxidant properties, since the thiol group of cysteine is a reducing agent and can be reversibly oxidized (GSSG) and reduced (GSH). GSH is maintained in the reduced form by the enzyme, glutathione reductase (GR), and functions by reducing other metabolites and enzyme systems. Glutathione peroxidase (GPx) catalyzes the oxidation of GSH to GSSG in the presence of ROS (Figure 1). GSSG can be converted back into GSH via GR and the conversion of NADPH to NADP+ [51]. Proton-translocating mitochondrial nicotinamide nucleotide transhydrogenase (NNT) catalyzes the reduction of NADP+ by NADH and is an important source of NADPH, as has been demonstrated using *nnt-1* deletion mutants [52]. Glutathione S-transferases (GSTs) can catalyze the conversion of GSH to GS−, which can then form complexes with various xenobiotics to facilitate excretion [53]. These enzymes are found at particularly high levels in the liver, and GSH is typically the most abundant sulfhydryl-containing compound in cells. Additionally, GSH is known to offer protection against metal-generated ROS by binding free radicals [54]. GSHs have been reported to increase, decrease, or remain constant after exposure to metals [55–57]. The GSH system is found in animals, plants, and microorganisms. *C. elegans* express approximately 50 GSTs [58]. Additionally, phytochelatins (PCs), a family of metal-inducible peptides synthesized enzymatically from GSH by PC synthase (PCS) in the presence of heavy metal ions, have been identified in *C. elegans* [34]. Although PCs are synthesized from GSH, they are broadly classified as class III metallothioneins and have been shown to be important in the detoxification of heavy metals [34]. 

### 2.1. Characterization of *C. elegans* GSH and Interaction with Metals

Liao and Yu [35] investigated the involvement of the GSH system in response to inorganic arsenic exposure. Results confirmed that oxidative stress plays a role in arsenic-induced toxicity by mutating glutamylcysteine synthetase (GCS) (gcs-1), the rate-limiting enzyme in GSH synthesis, in worms. The *gcs-1* loss-of-function strain demonstrated hypersensitivity to arsenic exposure in lethality testing, as compared to wild-type animals, an effect that was rescued by the addition of GSH to the medium, indicating that these enzymes are crucial for mediating arsenic-induced toxicity [35]. 

Furthermore, Helmcke and Aschner [39] reported a significant increase in fluorescence in a *gst-4*::GFP strain following both acute and chronic exposure to MeHg [39]. It was also demonstrated that knockout *gst-4* worms did not display greater sensitivity to MeHg than did N2 wild-type worms. GSH levels were found to be increased in worms subjected to acute exposure, whereas worms subjected to chronic exposure exhibited depleted levels of GSH. In their hormetic model, Helmcke and Aschner demonstrated an increase in *gst-4*::GFP expression after low concentration, acute exposure, a finding which indicates that *gst-4* may be involved in the hormetic response to MeHg [39]. Taken together, the data from these studies suggest that *gst-4* contributes to the response to MeHg exposure, but that knockdown of this gene does not affect with overall lethality. 

In a study examining the role of PCS in the elimination of cadmium, Vatamaniuk and colleagues [36] reported that the *C. elegans pcs-1* gene encodes a functional PC synthase critical for heavy metal tolerance. Using double stranded RNAi against *pcs-1, *this group showed that, although the progeny of worms injected with the dsRNAi and exposed to cadmium (5–25 *μ*M) managed to reach adulthood, these worms were small, necrotic, sterile, and had a much shorter lifespan than did the wild-type controls [36]. After being exposed to 50 and 100 *μ*M concentrations of cadmium, the *pcs-1* worms arrested at the L2–L4 stage were necrotic and died by day 6. The results were definitively dependent on the presence of cadmium, because *pcs-1-*deficient worms, in the absence of cadmium exposure, responded identically to the wild-type controls [36]. 

Hughes and colleagues [37] examined metabolic profiles following cadmium exposure in phytochelation synthase-1 (*pcs-1*) mutants. Results from these studies showed that the primary response to low levels of cadmium is the regulation of the transsulfuration pathway, due to the fact that cadmium exposure caused a decrease in cystathionine concentrations and an increase in phytochelation-2 and -3 [37]. These results were corroborated by additional studies which demonstrated that *pcs-1* mutants were an order of magnitude more sensitive to cadmium than were the metallothionein mutants. Furthermore, the MT-*pcs-1* triple mutant was found to display an additive sensitivity toward cadmium [37]. Significant findings are summarized in Table 2.

## 3. Metallothioneins

Metallothioneins (MTs) belong to a family of cysteine-rich low-molecular-weight metal-binding proteins (MW 3,500–14,000 Da) involved in metal detoxification and homeostasis [59]. MTs bind both metals of physiological importance such as copper and zinc, as well as xenobiotics including arsenic, cadmium, mercury, and silver. The binding of these metals occurs via the interaction of the cysteine residues with thiol groups. Cysteine residues represent approximately 30% of the amino acid content of metallothioneins. Binding of metals by MTs may be transient, as MTs are capable of rapidly releasing metal ions [60]. The protective roles of MTs can be ascribed to their three primary functions: (1) metal homeostasis, (2) heavy metal detoxification, and (3) protection from oxidative stress. Additionally, these proteins have been identified as contributors to the hormetic response [39, 61]. 

Mammals express four known metallothionein isoforms (MT-I, MT-II, MT-III, MT-IV) [62]. MT-I and MT-II are expressed in almost all tissues and have been best characterized with regard to their protection of the brain [62]. MT-III is especially enriched in the central nervous system, although its role has not yet been clearly defined [63]. MT-IV is most abundantly expressed in the stratified squamous epithelia [64–66]. MT expression has been shown to be induced under stressful cellular conditions such as exposure to cytokines, glucocorticoids, reactive oxygen species (ROS), and metal ions [67]. MTs can bind directly and sequester the toxicant; they also can provide protection by acting as antioxidants [59] (Figure 1). Further, MTs can limit apoptosis and promote the survival of mitochondrial dysfunctional cells by serving as highly efficient reducing elements against reactive oxygen species (ROS) [68].* C. elegans* contain two distinct isoforms of MTs, known as *mtl-1* and *mtl-2,* which can be induced in response to exposure to various metals [69]. 

### 3.1. Characterization of MTs and Their Interaction with Metals

Jiang and colleagues examined the effects of MTs on depleted uranium (DU) in *C. elegans* [12]. This group demonstrated concentration-dependent DU toxicity and protection by MTs. Results from their study showed that *mtl-1* was an important factor in uranium accumulation in *C. elegans* as knockouts displayed increased cellular accumulation [12]. 

In a study investigating lead and methylmercury toxicity, Ye and colleagues [38] demonstrated the involvement of MTs in affording a protective cross-adaptation response to neurobehavioral toxicity. This endpoint was assessed by observing behavioral alterations (head thrashing and body bending) in worms that were exposed during the L2 phase to either Pb or MeHg [38]. The study was conducted in conjunction with mild heat shock, wherein pretreatment of the larva with heat shock prevented the neurobehavioral deficits and the stress response at lower concentrations (50–100 *μ*M) but not at higher concentrations (200 *μ*M). Additionally, mild heat shock coupled with exposure to a low concentration of either metal was found to induce *mtl-1* and *mtl-2* promoter activity and GFP gene expression, results that were not observed in either the metal-exposed or heat-shocked cohort alone. Finally, the overexpression of *mtl-1* or *mtl-2* at the L2 stage was shown to significantly repress neurobehavioral toxicity, suggesting that the accumulation of MT protein is necessary to confer the protective response to the toxicant. 

Similarly, Helmcke and Aschner [39] reported that *mtl *knockouts displayed increased lethality upon exposure to MeHg. This group also demonstrated increases in *mtl-1*::GFP fluorescence in response to acute MeHg exposure at the L1 stage; however, chronic MeHg exposure produced no change in florescence [39]. Their results indicate that *mtl-1* is important in mediating MeHg toxicity and that the effects occur in a concentration- and time-dependent manner.

Meyer and colleagues [40] examined the aggregation of silver nanoparticles (AgNPs) in wild type and *mtl-2 C. elegans*. Results from these studies showed that the *mtl-2* strain displayed greater AgNP sensitivity than did the wild-type controls. AgNPs were internalized, and the observed toxicity was mediated by ionic silver [40]. These data indicate that there may be a differential preference for *mtl-1* over *mtl-2* depending on the particular metal to which an organism is exposed. 

In a 2004 study by Swain and colleagues [41], MTs were shown to play an important role in cadmium trafficking. Using GFP-expressing transgenes, MT-null alleles, and the RNAi knockdown of MTs, this group demonstrated that cadmium but not copper or zinc was able to influence a concentration-dependent, temporal transcription response. Both MT isoforms were found to be independent and not synergistic. Cadmium exposure caused a reduction in body size, generation time, brood size, and lifespan, effects that were magnified in the MT knockdown worms [41]. 

Hughes and colleagues [37] studied metabolic profiles using proton NMR spectroscopy and UPLC-MS following cadmium exposure in single and double *mtl* knockouts. Results showed that the metallothionein status did not influence the metabolic profile in cadmium-exposed or unexposed worms. The primary response to low levels of cadmium was the regulation of the transsulfuration pathway, due to the fact that cadmium exposure resulted in a decrease in cystathionine concentrations and an increase in phytochelation-2 and -3 [37]. These results were corroborated by data showing that *pcs-1* mutants (phytochelation synthase-1) were an order of magnitude more sensitive to cadmium than were MT mutants. Further, an additive sensitivity toward cadmium was observed in the MT-*pcs-1* triple mutant [37]. 

A study by Bofill and colleagues [42] examined zinc and cadmium toxicity; results indicated differential metal binding behavior for MT-1 as compared to MT-2. Specifically, the MT-1 isoform showed optimal behavior when binding Zn, and MT-2 showed optimal behavior when binding Cd. Accordingly, it was hypothesized that, due to its induction following Cd exposure, MT2 is primarily responsible for detoxification, whereas MT1 possesses some degree of constitutive expression and is, therefore, primarily involved in physiological metal metabolism (e.g. zinc) [42]. These findings were corroborated by additional studies which showed that MT-knockout worms exhibited significantly decreased levels of overall fitness after the knockout of MT1 than after MT2 knockout. Further, both MT isoforms displayed a clear preference for divalent metal ion binding as opposed to copper coordination, likely due to the presence of histidines in the MTs [42]. 

Using both *in vitro* and *in vivo* models, Zeitoun-Ghandour and colleagues [43] examined zinc and cadmium exposures and showed different roles for *mtl-1* and *mtl-2*. Both isoforms were expressed *in vitro* and were exposed to either Zn(II) or Cd(II). Their affinities and stoichiometries were measured, and both isoforms displayed equal zinc- binding ability; however, *mtl-2* had a higher affinity for Cd than did *mtl-1*. These experiments were repeated *in vivo* in *mtl-1*, *mtl-2,* and double knockouts following exposure to 340 *μ*M Zn or 25 *μ*M Cd. Zinc levels were found to be significantly increased in all knockout strains, but *mtl-1* knockout worms demonstrated the most acute level of sensitivity. However, cadmium accumulation was found to be the highest in the *mtl-2* knockout and double mutant strains. Additional studies assessed metal speciation, and results indicated that O-donating ligands play an important role in maintaining zinc levels, independent of metallothioneins status. Further, cadmium was shown to interact with thiol groups, and Cd speciation was significantly different in the *mtl-1* strain when compared with both the *mtl-2* strain and the double knockout strain, suggesting that the two MT isoforms have distinct *in vivo* roles [43]. The authors suggested that MTs are not functioning as metal storage proteins but, rather, are mediating the accumulation and excretion of metals. A follow-up study, showed *in vitro* evidence for the partitioning of zinc and cadmium with different metallothionein isoforms [70]. Employing electrospray ionization mass spectrometry (ESI-MS) to directly observe zinc and cadmium binding preferences, more cadmium ions were found to be preferentially bound to MT-2 than to MT-1; however, Cd^2+^ was shown to be capable of inducing both isoforms. Finally, partitioning was also demonstrated to be more effective at lower Cd : Zn ratios [70]. 

Using *daf-2* (insulin receptor-like protein) and *age-1* (phosphatidylinositol-3-OH kinase catalytic subunit) mutants, Barsyte et al. [44] examined the expression of MT genes under noninducing conditions and after exposure to cadmium and copper. They reported that MT-1 mRNA levels were significantly higher in *daf-2* mutants compared to both *age-1* mutants and wild-type worms under basal conditions. This study also assessed constitutive MT-1 expression and inducible MT-2 expression. Exposure to cadmium treatment resulted in a three-fold induction of MT-1 and a two-fold induction of MT-2 mRNA in *daf-2* mutants as compared to wild-type controls. Copper did not induce MT-1 or MT-2 mRNA expression in any of the strains tested [44]. 

Collectively, these studies show differential metal preferences for one MT isoform over another depending on the metal to which an organism is exposed. Most significantly, these studies indicate that the MTs play crucial roles in metal detoxification (Table 2). Indeed, MTs have been associated with a protective effect in cells under numerous states of disease and stress. 

Interestingly, serum MT levels of cancer patients are three times higher than those of control patients [71]. A study conducted in Denmark revealed the increased expression of MT-1 and MT-2 mRNA and protein in many human cancers such as breast, kidney, lung, nasopharynx, ovary, prostate, salivary gland, testes, urinary bladder, cervical endometrial skin carcinoma, melanoma, acute lymphoblastic leukemia, and pancreatic cancers [72]. This information is of particular import given the use of metals for the treatment of certain cancers, for example, arsenic as treatment for promyelocytic leukemia. It is interesting to postulate that higher levels of MTs may enhance the efficacy of metal therapeutic agents or, conversely, may lead to resistance to such therapies. Understanding the factors that modulate MT expression will allow for the improved understanding of metalloid toxicity and will provide more effective therapeutic approaches to metalloid-based chemotherapy.

## 4. Pumps and Transporters

There are a number of pumps and transporters that have been implicated in metal detoxification. These include ATP-binding cassette (ABC) transporters, such as the multidrug resistance-associated protein (MRP) as well as two members of the P-glycoprotein subfamily (PGP-1 and PGP-3), which have been shown to contribute to heavy metal tolerance through the use of *C. elegans* deletion mutants. In *C. elegans*, there are approximately 60 genes encoding ABC transporters, and these genes make up the largest family of transporters [73]. *C. elegans* have four MRP homologues [46] and fifteen Pgp homologues [73]. The Pgps are ubiquitously expressed and are most abundantly found in the apical membranes of the gut and in the excretory organs of the worm [74]. The specific functions of three of the nematode *pgps* have been identified. *pgp-2* is expressed in the intestine and is required for the acidification of lysosomes and lipid storage; *pgp-1* and *-3* contribute to heavy metal and drug resistance [46]. 

### 4.1. Characterization of Pumps and Transporters and Their Interaction with Metals

Tseng and colleagues [45] investigated the ArsA protein-mediated detoxification of the metalloids, As(III) and Sb(III). Bacterial ArsA ATPase is the catalytic component of an oxyanion pump that is responsible for resistance to arsenite and antimonite. In this study, wild-type and *asna-1*-mutant nematodes were evaluated for As and Sb response and toxicity. The *asna-1* gene of *C. elegans* was found to be stimulated by As(III); further, Sb(III) was determined to be crucial for establishing tolerance. Although these results occurred in response to As and Sb exposure, the ubiquity of the ArsA ATPase-dependent pathway has not been observed in other species or in response to other metals [45]. Moreover, the exact mechanism(s) of protection has not yet been elucidated. 

The role of multidrug resistance-associated protein (MRP) in arsenite and cadmium toxicity was explored in a study by Broeks and colleagues [46]. The targeted inactivation of *mrp-1* rendered the arsenite—and Cd^2+^—exposed worms incapable of recovering from temporary exposure to high arsenic and cadmium, whereas the wild-type controls were able to recover. Additionally, worms were also shown to be hypersensitive to arsenite and Cd^2+^ exposures when both *mrp-1* and *pgp-1* (P-glycoprotein-1) were deleted [46]. Lastly, no increased sensitivity in response to exposure to antimony was observed in *mrp-1*-deletion mutants as compared to wild-type controls [46].

Vatamaniuk and colleagues [47] characterized the half-molecule ABC transporter of the heavy metal tolerance family-1 (HMT-1) subfamily in response to cadmium exposure. The suppression of *hmt-1* expression by RNAi was shown to produce punctuate refractive inclusions within the vicinity of the nucleus of the intestinal epithelial cells upon exposure to toxic levels of cadmium [47]. Similarly, Schwartz and colleagues described the *C. elegans* HMT-1 following exposure to arsenic, copper, and cadmium. HMT-1 conferred tolerance in response to exposure to all three metals as shown by lethality testing following the knockdown of *hmt-1* [48]. 

Kurz and colleagues [49] demonstrated the three-fold induction of *pgp-5* following cadmium exposure. Results of this study showed that strong fluorescence was induced in the intestinal cells of *pgp-5*::GFP worms, where the GFP-encoding gene is under the control of the upstream *pgp-5* promoter [49]. Copper and zinc were also found to be capable of inducing *pgp-5* expression in these worms. Mutant *pgp-5* worms exhibited a developmental delay upon exposure to cadmium and copper. Accordingly, it was concluded that *pgp-5* is required for establishing full resistance to cadmium and copper. In addition, the RNAi knockdown of *tir-1*, an upstream component of the p38 MAPK pathway in the *pgp-5* transgenic reporter strain, was shown to significantly reduce *pgp-5* induction following exposure to cadmium. However, the double-stranded RNA knockdown of ERK (*mpk-1*) and JNK (*med-1* and *kgb-1*) did not affect the induction of *pgp-5* in response to cadmium exposure [49]. 

Au and colleagues [50] studied the divalent-metal transporter (DMT1) following exposure to manganese. The DMT1-like family of proteins has been shown to regulate manganese and iron in the cell. The deletion of the three worm DMT1-like genes resulted in differential effects on manganese toxicity. The deletion of *smf-1* and *smf-3* increased Mn tolerance, whereas the deletion of *smf-2* increased Mn sensitivity [50]. Significant findings are summarized in Table 2.

## 5. Heat Shock Proteins

Heat shock proteins (HSPs) are cytosolic molecular chaperones. HSPs promote the refolding and repair of denatured proteins and facilitate protein synthesis upon activation by cellular stress [75, 76]. HSPs, particularly those in the HSP70 family, have also been shown to participate in the hormetic response [39]. HSP70s are ATP-binding proteins that convert ATP to ADP and bind to peptides, thereby, inactivating them and preventing aggregation (Figure 1). Oxidative stress can cause a reduction in cellular ATP levels [77]. Decreased levels of ATP result in the continued prevention of the aggregation of damaged proteins [78]. The functions of the HSP70 products are mediated by the conserved N-terminal ATPase and the C-terminal peptide-binding region [79]. The human and *C. elegans* HSP70 genes have a high degree of homology and share a conserved core “ATPase” structure [79]. The Hsp16 family of stress proteins is produced in *C. elegans* only under stress conditions [80–82].

### 5.1. Characterization of HSPs and Their Interaction with Metals

In a study examining the effects of MeHg exposure, hsp-4::GFP was measured immediately following the treatment of L1 worms for 30 minutes and L4 worms for 15 hours with this toxicant [39]. After 30 minutes of acute exposure to MeHg, the fluorescence of *hsp-*4::GFP remained unaltered. However, in L4 worms chronically exposed to MeHg for 15 hours, *hsp-4*::GFP was induced. At the same time point in the chronic treatment paradigm, a four-fold increase in *gst-4* fluorescence was detected, but there were no changes in either *mtl-1* or *mtl-2::*GFP expression [39]. 

Jones and Candido [18] exposed nematodes to cadmium or mercury and measured feeding behavior. For these studies, transgenic lines containing the promoter sequence for hsp16 genes which regulate the production of *E. coliβ*-galactosidase were used. Accordingly, to measure stress, levels of this protein were assessed. Results showed that cadmium inhibited feeding behavior significantly but not completely, as a minimal rate of feeding continued at high cadmium concentrations. Further, exposure to cadmium (1 ppm) induced a detectable production of *β*-galactosidase without inhibiting feeding behavior. The stress response was induced at a concentration of cadmium that was ten times lower than the LC50. Mercury also was shown to inhibit feeding at concentrations similar to those necessary for the induction of a stress response; however, the difference in this instance was less than two fold. Mercury also did not entirely inhibit feeding behavior [18]. Significant findings are summarized in Table 2.

## 6. Conclusion

The use of *C. elegans* as an experimental model has produced considerable insight and valuable information regarding the multiple and varied processes of metal detoxification. Conclusive biochemical evidence has indicated that different metals are not handled in the same capacity. Many metalloregulatory proteins exhibit selectivity toward their target metal ions. The selectivity and sensitivity of each of these proteins is highlighted in the large body of accumulated research on different metal toxicities as well as various systems of metal detoxification. However, the overall mechanisms, temporal activation, and interplay between different cell detoxification systems remain elusive. Future studies are necessary in order to enhance our understanding of the complex interplay of multiple-cell detoxification systems in response to exposure to different metals. The *C. elegans* model system will be critical for these investigations, as knockouts are easily generated and provide a wealth of information about metal detoxification in a genetically retractable, inexpensive, and *in vivo* model.

## Figures and Tables

**Table 1:** Points to consider when using *C. elegans*.

(i) *Metal concentrations*: Must be measured in worms because of potential differences in uptake due to the worm cuticle (versus ingestion through the pharynx). In addition to concentrations, attention should be paid to the specific metallic salt under consideration as well as speciation that may occur following exposure.	
(ii) *Age matching:* May be necessary depending on the toxic endpoint measured due to potential for developmental delay in knockout strains.	
(iii) *Dauer stage:* An alternative developmental stage when the larva goes into a type of stasis and becomes lethargic, ceases pharyngeal pumping, synthesizes a new cuticle under the old, and can survive harsh conditions [16].	
(iv) *Source of exposure*: *C. elegans *exhibit avoidance behavior and have been shown to avoid certain volatile compounds [29] as well as high concentrations of salts and sugars [30, 31].	
(v) *Medium considerations*: *C. elegans* exhibit a wide pH range tolerance and thus can be exploited to measure alterations in toxicity of metals following pH elevation [32]. Differential effects of soil versus aquatic medium have also been documented [33].	

**Table 2:** Summary of significant findings.

Metals	Effects Observed	Reference
Glutatione		

Arsenic	Nematodes with loss of glutamylcysteine synthetase (*gsc-1*) demonstrated hypersensitivity to arsenic exposure in lethality testing. Effect was rescued by the addition of GSH to medium.	[34]

Methylmercury	Significant increase in fluorescence of gst-4::GFP following MeHg exposure. Knockout *gst-4* worms not more sensitive than wildtype. GSH levels increased following acute exposure; chronic exposure depleted levels of GSH. GSH found to regulate the hormetic response.	[35]

Cadmium	Low Cd exposure in phytochelation synthase-1 (*pcs-1*) RNAi worms resulted in worms that were small, necrotic, sterile, and had a shorter lifespan. Following higher concentrations of Cd, *pcs-1* worms arrested at L2–L4 stage were necrotic and died.	[36]

Cadmium	Primary response to low levels of cadmium is the regulation of the transsulfuration pathway due to decreases in cystathionine concentrations and increases in phytochelation-2 and -3. MT-pcs-1 triple mutants showed added sensitivity.	[37]

Metallothioneins		

Depleted uranium	Concentration-dependent DU toxicity and protection by MTs. Mtl-1 knockouts displayed increased cellular accumulation of DU.	[12]

Lead and methylmercury	Pretreatment of larva with heat shock prevented the neurobehavioral deficits and the stress response at lower concentrations (50–100 *μ*M) but not at higher concentrations (200 *μ*M). Mild heat shock and low concentration of either metal found to induce *mtl-1* and *mtl-2* promoter activity and GFP expression. Overexpression of *mtl-1* or *mtl-2* at L2 stage significantly repressed neurobehavioral toxicity.	[38]

Methylmercury	Mtl knockouts displayed increased lethality upon exposure to MeHg. Increases in *mtl-1* following acute MeHg exposure at L1 stage but no change following chronic exposure.	[39]

Silver Nanoparticles	Mtl-2 strain displayed greater AgNP sensitivity than wildtype. Toxicity mediated by ionic silver.	[40]

Cadmium	MT isoforms found to be independent and not synergistic. Cadmium but not copper or zinc was able to influence a concentration-dependent, temporal transcription response.	[41]

Cadmium	Metallothionein status did not influence the metabolic profile in cadmium-exposed or -unexposed worms. Primary response was the regulation of the transsulfuration pathway.	[37]

Zinc and cadmium	Differential metal binding behavior for MT-1 compared to MT-2. MT-1 had optimal behavior when binding Zn, MT-2 optimal behavior when binding Cd.	[42]

Zinc and cadmium	Zinc levels significantly increased in *mtl-1*, *mtl-2,* and double knockouts, *mtl-1* knockout worms demonstrating the most acute level of sensitivity. Cd accumulation found to be highest in *mtl-2* and double mutant strains.	[43]

Cadmium and copper	MT-1 mRNA levels significantly higher in *daf-2* mutants compared to *age-1* mutants and wild-type worms under basal conditions. Cd treatment resulted in 3-fold induction of MT-1 and 2-fold induction of MT-2 mRNA in *daf-2* mutants compared to wild-type controls. Copper did not induce expression in any of the strains tested.	[44]

Pumps and Transporters		

Arsenite and antimonite	ArsA ATPase (*asna-1*) gene stimulated by As (III) and Sb (III) crucial for establishing tolerance.	[45]

Arsenite and Cadmium Antimony	Inactivation of *mrp-1* rendered As and Cd exposed worms incapable of recovering from temporary exposure to high As and Cd, wildtype worms were able to recover. Worms were hypersensitive to As and Cd exposures when both *mrp-1* and *pgp-1* were deleted. No increased sensitivity in response to antimony observed in *mrp-1* deletion mutants compared to wild types.	[46]

Cadmium	Suppression of *hmt-1* (half-molecule ABC transporter of the heavy metal tolerance family-1) by RNAi shown to produce inclusions within the nucleus of the intestinal epithelial cells upon exposure to toxic levels of Cd.	[47]

Arsenic, copper, and cadmium	HMT1—conferred tolerance in response to exposure to all three metals revealed through lethality testing following knockdown of *hmt-1*.	[48]

Cadmium	Three-fold induction of *pgp-5* following Cd exposure. Copper and zinc also found to be capable of inducing *pgp-5* expression. Mutant *pgp-5* worms showed developmental delay following Cd and Cu exposure.	[49]

Manganese	Deletion of the three DMT-1-like (divalent-metal transporter) genes resulted in differential effects. *smf-1* and *-3* increased Mn tolerance, and *smf-2* increased Mn sensitivity.	[50]

Heat Shock Proteins		

Methylmercury	Following 30 minute exposure to acute MeHg, *hsp-4* was unaltered. Hsp-4 induced in L4 worms chronically exposed to MeHg for 15 hours.	[39]

Cadmium and mercury	Cd-inhibited feeding behavior significantly but not completely. Exposure to 1 ppm Cd induced hsp16 genes. Hg also did not entirely inhibit feeding behavior and was shown to inhibit feeding at concentrations similar to those necessary for the induction of a stress response.	[18]
